# Prediction of Early Mortality in Esophageal Cancer Patients with Liver Metastasis Using Machine Learning Approaches

**DOI:** 10.3390/life14111437

**Published:** 2024-11-06

**Authors:** Yongxin Sheng, Liyuan Zhang, Zuhai Hu, Bin Peng

**Affiliations:** School of Public Health, Chongqing Medical University, Chongqing 400016, China; 2022110589@stu.cqmu.edu.cn (Y.S.); 2022120860@stu.cqmu.edu.cn (L.Z.); 2022120774@stu.cqmu.edu.cn (Z.H.)

**Keywords:** esophageal carcinoma, liver metastasis, early death, predictive model

## Abstract

Patients with esophageal cancer liver metastasis face a high risk of early mortality, making accurate prediction crucial for guiding clinical decisions. However, effective predictive tools are currently limited. In this study, we used clinicopathological data from 1897 patients diagnosed with esophageal cancer liver metastasis between 2010 and 2020, which were sourced from the SEER database. Prognostic factors were identified using univariate and multivariate logistic regression, and seven machine learning models, including extreme gradient boosting (XGBoost) and support vector machine (SVM), were developed to predict early mortality. The models were evaluated using Receiver Operating Characteristic (ROC) curves, calibration curves, decision curve analysis (DCA), and F1 scores. Results showed that 40% of patients experienced all-cause early mortality and 38% had cancer-specific early mortality. Key predictors of early mortality included age, location, chemotherapy, and lung metastasis. Among the models, XGBoost performed best in predicting all-cause early mortality, while SVM excelled in predicting cancer-specific early mortality. These findings demonstrate that machine learning models, particularly XGBoost and SVM, can serve as valuable tools for predicting early mortality in patients with esophageal cancer liver metastasis, aiding clinical decision making.

## 1. Introduction

Esophageal carcinoma (EC) is a major malignant tumor that poses a serious threat to human health worldwide with a persistently high incidence rate [1]. According to 2020 statistics, there were over 600,000 new cases of esophageal cancer worldwide with nearly 540,000 deaths [2]. Despite recent advancements in the diagnosis and treatment of EC, the global 5-year survival rate for EC patients is only 15% to 20% [3]. EC often develops insidiously, with a lack of obvious symptoms in the early stages, leading to the majority of patients being diagnosed at the advanced stages of the disease. More than half of EC patients already have lymph node or distant organ metastases at the time of initial diagnosis [4]. Distant organ metastasis is one of the major challenges in the treatment of EC and a leading cause of high mortality. For patients with esophageal cancer liver metastasis (esophageal cancer liver metastasis, ECLM), the 5-year survival rate is less than 5% [5].

A study on the metastasis patterns of EC reported that in patients initially diagnosed with stage IV EC, the liver is the most common site of metastasis, accounting for 33.4%, which is followed by distant lymph nodes (26.6%), lungs (20.5%), bones (15.7%), and brain (3.8%) [6]. Notably, distant metastasis significantly impacts patient survival time with research indicating that the median survival time for ECLM patients is only 5 months (IQR: 2–10 months) [7]. The widespread application of machine learning algorithms has brought new perspectives to the improvement of cancer prediction models [8]. Machine learning methods, including random forest (RF), support vector machine (SVM), decision tree (DT), and Bayesian network (BN), have been widely used in cancer diagnosis and prognosis research. These advanced algorithms can explore nonlinear relationships between variables and provide methods for calculating risk probabilities in different datasets, demonstrating higher predictive accuracy [9].

In patients with liver metastatic esophageal carcinoma (ECLM), accurate prediction of survival time is crucial for formulating effective treatment strategies. Currently, there is no standard treatment for ECLM, and current treatments mainly include chemotherapy, radiotherapy, and best supportive care, which are aimed at alleviating symptoms and improving quality of life [10]. Although some small-scale studies have shown that surgical treatment can improve survival rates in certain cases [11,12], surgery is usually not the first choice because inappropriate surgical intervention may increase the patient’s burden or even accelerate death [13]. In this context, clinicians need to formulate individualized treatment plans for ECLM patients after comprehensively assessing treatment risks and patient quality of life. Therefore, accurately predicting the survival time of patients becomes particularly important, as it not only helps doctors choose the most appropriate treatment methods but also improves the overall prognosis and quality of life of patients.

Patients with metastatic esophageal carcinoma have poor clinical outcomes and a high rate of early death [14]. Early death is typically defined as death occurring within 3 months of initial diagnosis regardless of whether the patient has received treatment [15]. Although many studies have focused on the overall survival of ECLM patients, there is limited research on early death in ECLM patients, and some studies suggest that the factors influencing early and late survival may differ [16]. The accurate prediction of early death can help clinicians identify high-risk patients at an early stage, allowing for timely adjustments in treatment strategies to avoid unnecessary invasive procedures and maximize quality of life.

In recent years, machine learning methods have shown excellent performance in predicting early death in metastatic cancer patients. For example, in a study of bone metastatic breast cancer patients, Xiong et al. [17]. developed a machine learning-based model to predict early death risk. The study involved 16,189 patients and used various machine learning algorithms, including random forest (RF), support vector machine (SVM), and extreme gradient boosting (XGBoost). The results showed that the gradient boosting machine model performed best in terms of discrimination and calibration, demonstrating excellent overall predictive performance. This study highlighted the potential of machine learning in handling large-scale medical datasets and predicting patient outcomes as well as the relative strengths and weaknesses of different algorithms in practical applications.

In a study on early death risk in lung cancer patients with bone metastases, Cui et al. [18] analyzed the clinical characteristics of 19,887 patients using various machine learning algorithms with the gradient boosting machine model performing best and achieving an AUC of 0.82. Additionally, the study used the LIME method to reveal the contribution of each variable to the predictions. However, the study primarily focused on global explanations and did not provide detailed local explanations.

In the study of metastatic gastric cancer patients, Zhu et al. [19] developed a predictive nomogram and an online probability calculator for early death. The study utilized data from the SEER database and multi-center data from China, integrating various variables including patient demographics, tumor characteristics, and treatment information. The model was internally and externally validated with AUC values greater than 0.70 across different datasets, indicating high predictive accuracy and stability. However, the study mainly relied on traditional statistical methods, which may be less effective in handling complex nonlinear relationships.

Therefore, this study will leverage the SEER database and apply machine learning algorithms to gain a deeper understanding of the risk factors for early death in ECLM patients and develop an optimal predictive model. By combining global and local explanations, we aim to enhance the model’s credibility in practical applications. This will help clinicians identify patients with shorter survival times in a timely manner and develop targeted treatment plans with the goal of extending patient survival, improving quality of life, and avoiding unnecessary radical treatments, reducing the economic burden on patients, society, and families.

## 2. Methods

### 2.1. Data Source and Participants

The data used in this study were obtained from the Surveillance, Epidemiology, and End Results (SEER) database, which includes cancer registry data from 17 states in the United States, covering approximately 26.5% of the US population. The database is updated annually and serves as an authoritative source of cancer data in the United States [20]. The database is publicly accessible https://seer.cancer.gov (accessed on 28 March 2024), and data for this study were downloaded using SEER*stat 8.4.3. This study adhered to the ethical standards of the Declaration of Helsinki. Since SEER began collecting metastasis information in 2010, this study included patients diagnosed with liver metastatic esophageal carcinoma between 2010 and 2020.

**Figure 1:**
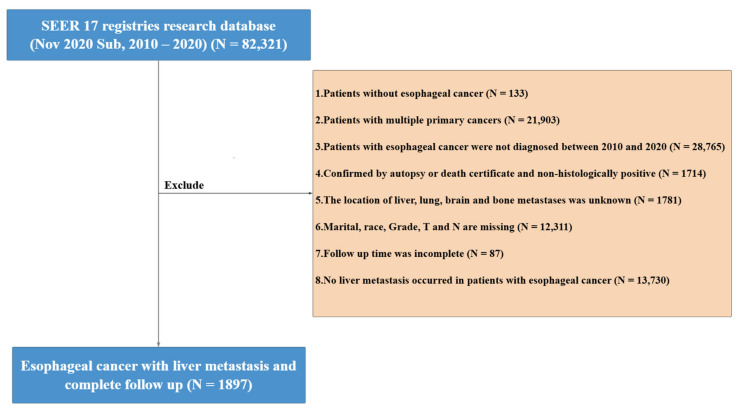
Esophageal cancer liver metastasis (ELCM) flowchart of patient selection.

Inclusion criteria for this study were (1) tumor site coded as esophageal cancer according to ICD-O-3 (International Classification of Diseases for Oncology); (2) behavior code as malignant; (3) diagnosis year between 2010 and 2020; and (4) patients aged 18 years or older. Exclusion criteria were (1) patients diagnosed with multiple primary cancers; (2) diagnosis confirmed through death certificate or autopsy report; (3) unknown metastasis site information; and (4) uncertain clinical data values. The flowchart of the study population selection is shown in Figure 1.

### 2.2. Feature Selection

Patient characteristics were extracted, including demographic information, tumor information, metastasis information, and clinical treatment interventions. Demographic information included age, gender, race, and marital status. Tumor information included primary tumor site, histological type, tumor T stage, N stage, tumor size, and tumor grade. Metastasis information included liver metastasis, brain metastasis, lung metastasis, and bone metastasis. Clinical treatment interventions included surgery, radiotherapy, chemotherapy, and the time from diagnosis to treatment (Timefdtt, month). The optimal cut-off value for age as a continuous variable was determined using X-tile software (Version 3.6.1, Yale University, New Haven, CT, USA). X-tile tested various truncation values, and the value with the smallest *p*-value was selected as the best threshold for the analysis [21]. Variables with more than 20% missing data, such as tumor size, were excluded, resulting in a final inclusion of 16 variables.

To simplify the model and extract meaningful variables, univariate and multivariate logistic regression analyses were performed to select features, identifying independent risk factors for liver metastasis and early death and establishing machine learning (ML) prediction models.

### 2.3. Model Development and Validation

The dataset was divided into a training set (75%) and a validation set (25%). Clinically meaningful and statistically significant variables were selected through logistic regression in the training set and then used to construct and optimize a range of machine learning models, including logistic regression (LR), k-nearest neighbors (KNN), support vector machine (SVM), random forest (RF), extreme gradient boosting (XGBoost), light gradient boosting machine (LightGBM), and decision tree (DT). All model training and hyperparameter optimization were conducted exclusively on the training set to prevent information leakage and ensure robust evaluation.

To enhance model effectiveness and reduce the risk of overfitting, all models used grid search during training to identify the optimal hyperparameter combinations (Supplementary Materials Tables S1 and S2) with 10-fold cross-validation employed to ensure the robustness of parameter selection [22,23]. The Area Under the Curve (AUC), a widely accepted classification metric, was used as the objective for hyperparameter optimization. In the KNN model, the optimal number of neighbors k was determined to improve classification accuracy. For the SVM model, the penalty parameter C was adjusted, and the most suitable kernel function (linear, polynomial, or radial basis function) was selected. For the RF model, the primary optimized parameters included the number of trees, the maximum number of features at each split, and the minimum leaf node size. The XGBoost and LightGBM models were tuned for learning rate and tree depth to balance convergence speed and generalization ability. Hyperparameter optimization for the DT model focused on the maximum tree depth and the choice of splitting criterion to control model complexity and prevent overfitting [24].

Once the model training and optimization were completed, an independent validation set was used to evaluate its predictive performance. Performance metrics included sensitivity (the ability to correctly identify positive cases), specificity (the ability to correctly identify negative cases), accuracy, precision, AUC (area under the ROC curve), and F1 score, providing a comprehensive evaluation of the model’s classification performance [25]. Precision–recall (PR) curves were constructed to assess model performance at different thresholds, allowing for insights into precision at varying recall rates. Calibration curves were used to assess the consistency between predicted probabilities and actual outcomes, while decision curve analysis (DCA) was conducted to evaluate the clinical utility of the model by calculating the net benefit across a range of threshold probabilities.

### 2.4. Model Interpretability and Feature Importance

This study enhances the interpretability of the model by demonstrating the relative importance of different variables in predicting liver metastasis and early death in esophageal cancer patients. We used the DALEX package for comprehensive analysis, which not only displayed the global importance of variables but also provided local explanations [26]. By performing single-sample prediction breakdowns, we specifically showed how each variable affects individual prediction results. This approach helps clinicians better understand and apply the model, providing strong support for personalized treatment.

### 2.5. Statistical Analysis

All variables in the dataset were categorized prior to analysis. Categorical variables were expressed as frequency (%). Comparisons between groups were conducted using chi-square tests or Fisher’s exact tests as appropriate. All statistical analyses were performed using R software (version 4.4.0; https://www.R-project.org (accessed on 20 April 2024)). A significance level of 0.05 (two-sided test) was set for all analyses.

## 3. Results

### 3.1. Demographic and Clinical Characteristics of Patients with Liver Metastasis of Esophageal Cancer

Based on strict inclusion and exclusion criteria, among the 1897 ECLM patients included from 2010 to 2020, 759 patients (40%) experienced early death, and 717 patients (38%) experienced cancer-specific early death. According to the analysis results from X-tile software, the optimal cutoff points for age were 58 and 75 years (Supplementary Materials Figure S1). The majority of ECLM patients were aged 59–75 years (54.5%), married (59.1%), of white race (88.8%), male (85.8%), with lesions most commonly located in the lower third of the esophagus (74.3%), and they predominantly had esophageal adenocarcinoma (77.3%). Only a very small proportion of patients underwent surgical treatment (1.8%), a minority received radiotherapy (34.3%), and most patients tended to receive chemotherapy (67.7%), as shown in Table 1.

It is evident that ECLM patients who were over 75 years old, married, white, and male had higher early death rates. Additionally, surgery, radiotherapy, and chemotherapy significantly reduced the early death rates of ECLM patients. According to the variance inflation factor and variable correlation heatmap (pairwise correlation), there was no serious multicollinearity problem in this study (Supplementary Materials Figures S2 and S3).

### 3.2. Risk Factors for Early Death from Liver Metastasis of Esophageal Cancer

**Figure 2:**
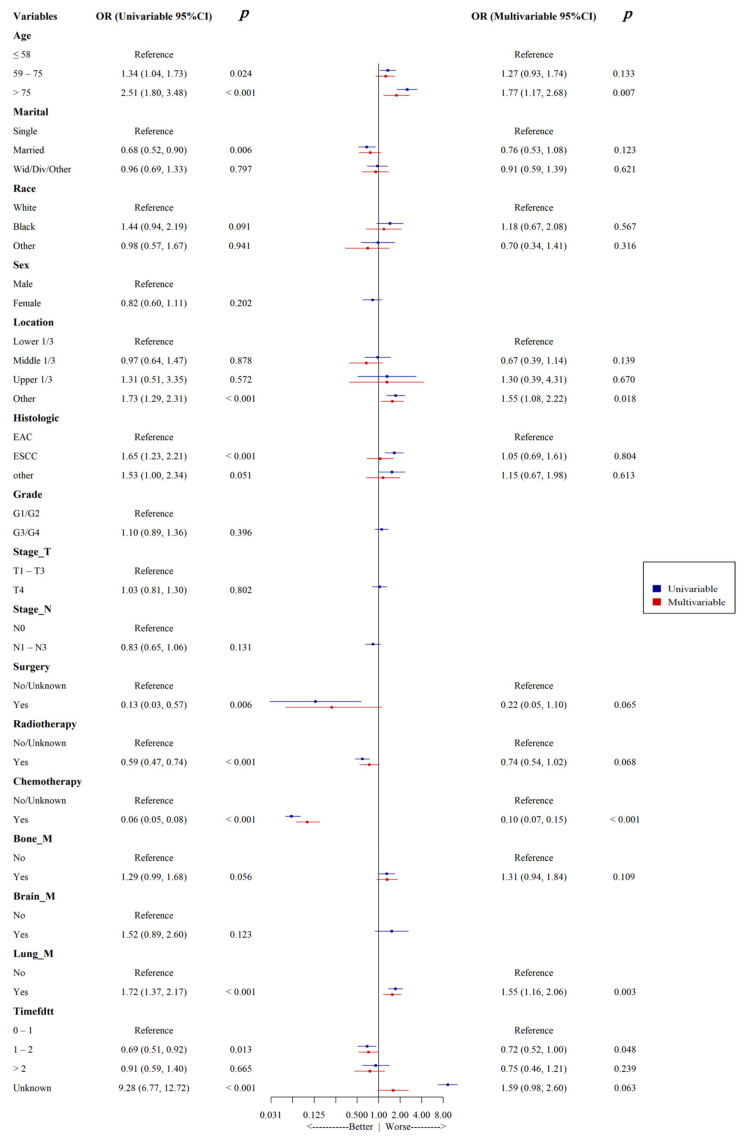
Univariate and multivariate logistic forest plots for all-cause early death in ECLM patients.

**Figure 3:**
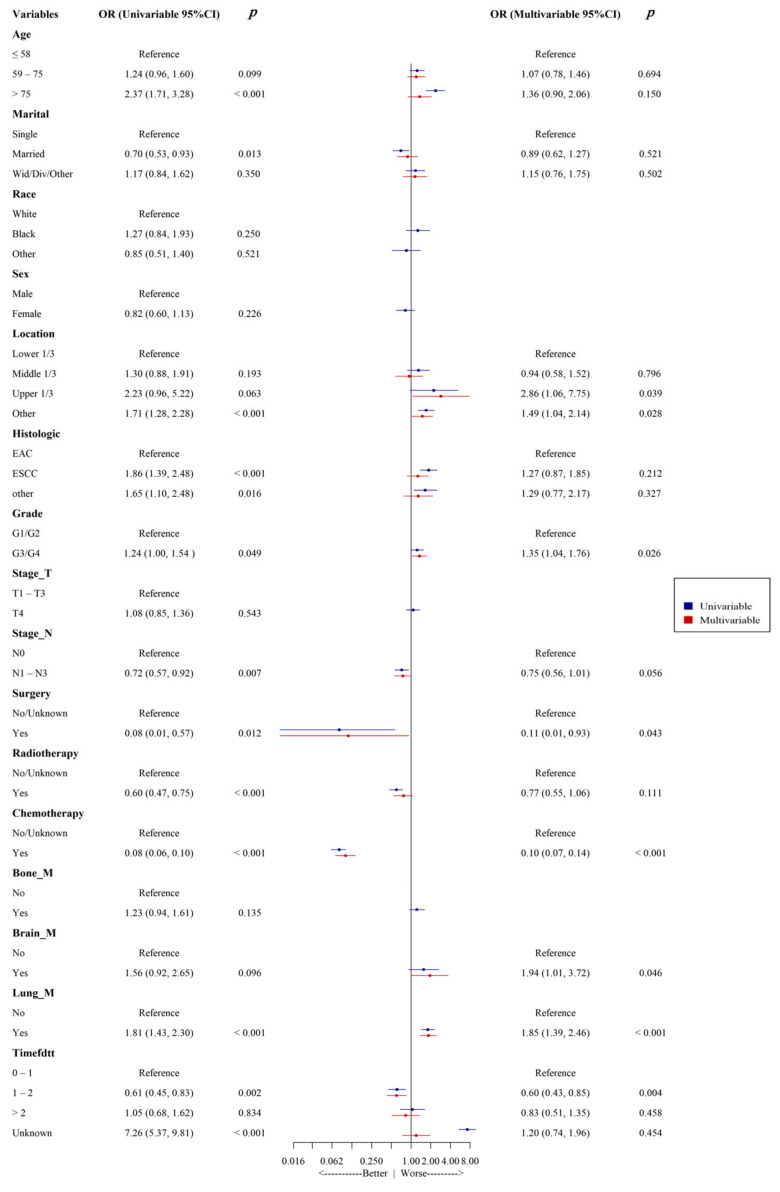
Univariate and multivariate logistic analysis forest plots for patient-specific early death in ECLM.

Univariate and multivariate logistic regression analyses were used to identify risk factors associated with all-cause early death in patients with esophageal cancer liver metastasis. The results (Figure 2) show that age, primary tumor site, chemotherapy, lung metastasis, and time from diagnosis to treatment were independent risk factors for all-cause early death in patients with esophageal cancer liver metastasis (all *p* < 0.05). Furthermore, primary tumor site, tumor grade, surgical treatment, chemotherapy, brain metastasis, lung metastasis, and time from diagnosis to treatment were independent risk factors for cancer-specific early death in these patients (all *p* < 0.05) (Figure 3).

### 3.3. Construction of Early Death Prediction Model for Patients with Liver Metastasis of Esophageal Cancer

**Figure 4:**
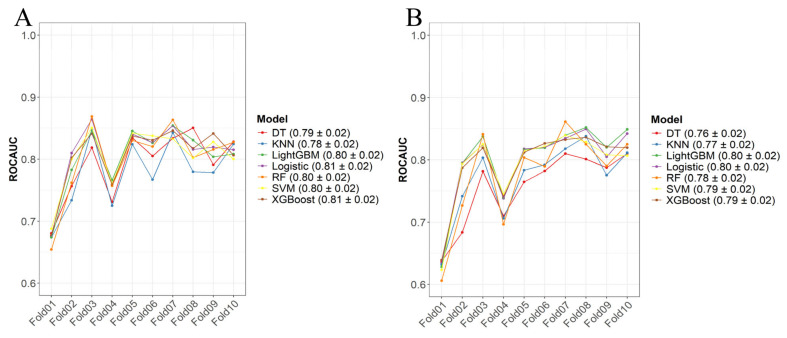
Ten-fold cross-verification in training sets. (**A**) All-cause early death model, (**B**) tumor-specific early death model.

Based on the independent predictive variables identified through univariate and multivariate logistic regression, seven early mortality prediction models for patients with esophageal cancer liver metastasis (ECLM) were constructed in the training set using LR, RF, XGBoost, LightGBM, DT, SVM, and KNN. The models were trained using ten-fold cross-validation with optimal parameters determined through grid search. The overall performance of the early mortality prediction models in the training set was good (Figure 4) with average AUC > 0.75 for both all-cause and cancer-specific early mortality models. The all-cause early mortality prediction models had the highest average AUC for LR and XGBoost (both 0.81, Std = 0.02), while the cancer-specific early mortality prediction models had the highest average AUC for LR and LightGBM (both 0.80, Std = 0.02).

### 3.4. Validation of Early Death Prediction Model in Patients with Liver Metastasis of Esophageal Cancer

**Figure 5:**
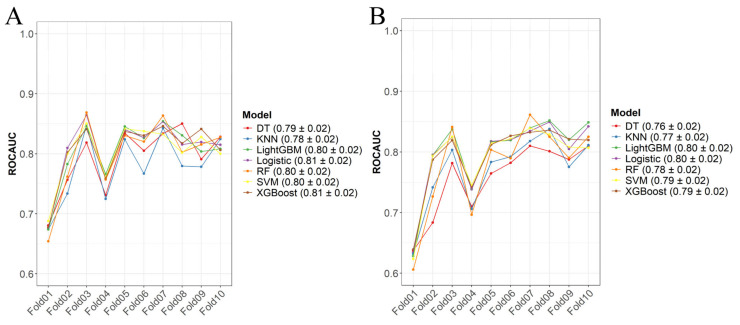
Ten-fold cross-verification in validation sets. (**A**) All-cause early death model, (**B**) tumor-specific early death model.

**Figure 6:**
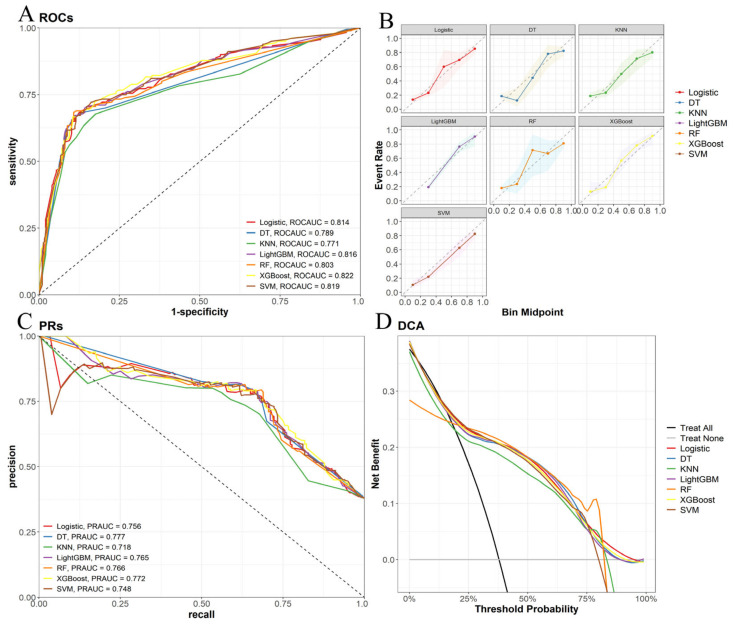
Validation of the all-cause to early death model. (**A**) Receiver operating characteristic (ROC) curve, (**B**) calibration curve, (**C**) accuracy–recall rate (PR) curve, (**D**) decision curve analysis.

In the validation set for all-cause early death, the results of the ten-fold cross-validation are shown in Figure 5A. The ROC curve (Figure 6A) shows that the XGBoost model has the highest AUC of 0.822, indicating the best performance in distinguishing between positive and negative samples. The calibration curve (Figure 6B) shows that the XGBoost and LightGBM models have better calibration compared to other models. The precision–recall (PR) curve (Figure 6C) shows that the DT model has the highest PR_AUC of 0.777, indicating that it maintains a high precision rate while keeping a high recall rate, with the XGBoost’s PR_AUC being 0.772, also performing well. The DCA curve (Figure 6D) shows the net benefit of each model under different thresholds, indicating that the LR and XGBoost models have a higher net benefit over most threshold ranges, proving their effectiveness in practical applications. The F1 score is an important comprehensive indicator of a model’s performance in classifying positive and negative samples. The XGBoost model has an F1 score of 0.725, sensitivity of 0.667, accuracy of 0.895, precision of 0.795, and accuracy of 0.809, which are all at the highest levels (Table 2). Although the RF model has the highest sensitivity (0.700), and the DT model performs well in PR_AUC (0.777), considering all indicators, the XGBoost model performs the best.

**Figure 7:**
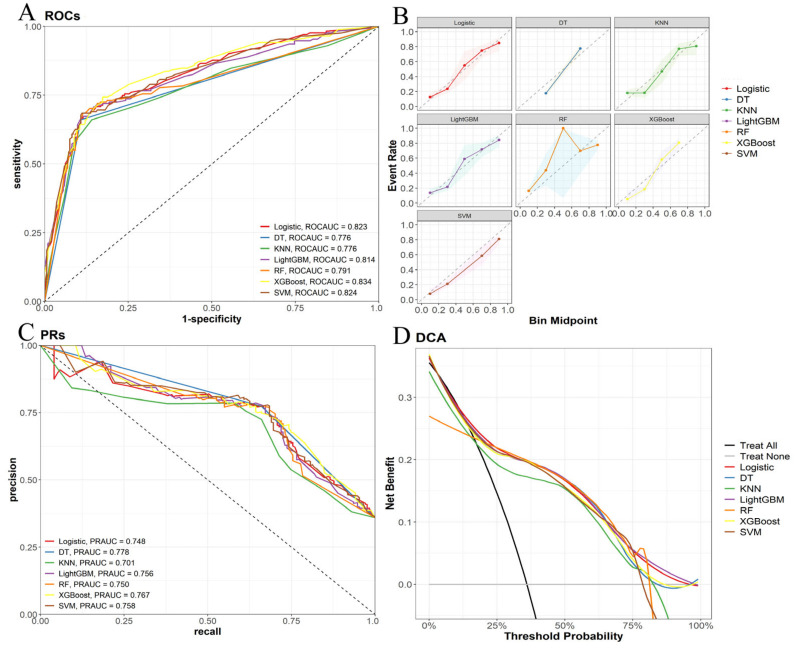
Validation of specific early death models. (**A**) Receiver operating characteristic (ROC) curve, (**B**) calibration curve, (**C**) accuracy–recall rate (PR) curve, (**D**) decision curve analysis.

In the validation set for cancer-specific early death, the results of the ten-fold cross-validation are shown in Figure 5B. The ROC curve (Figure 7A) shows that the XGBoost model has the highest AUC of 0.832, which is followed by the SVM model with an AUC of 0.824. The calibration curve (Figure 7B) shows that the LR and KNN models have the least deviation from the diagonal, indicating accurate prediction probabilities. The DT model has the highest PR_AUC of 0.778, while the XGBoost’s PR_AUC is 0.764, also performing well (Figure 7C). The DCA curve (Figure 7D) shows that the LR and LightGBM models have a higher net benefit over most threshold ranges. Additionally, the SVM model performs well in accuracy (0.811), sensitivity (0.684), specificity (0.882), and F1 score (0.722) (Table 3). Although the DT model performs best in PR_AUC (0.778) and the XGBoost model performs best in ROC_AUC (0.824), a comprehensive analysis shows that the SVM model performs best in balancing various performance metrics, especially in key indicators such as F1 score and accuracy.

### 3.5. Optimal Models Interpretability and Feature Importance

**Figure 8:**
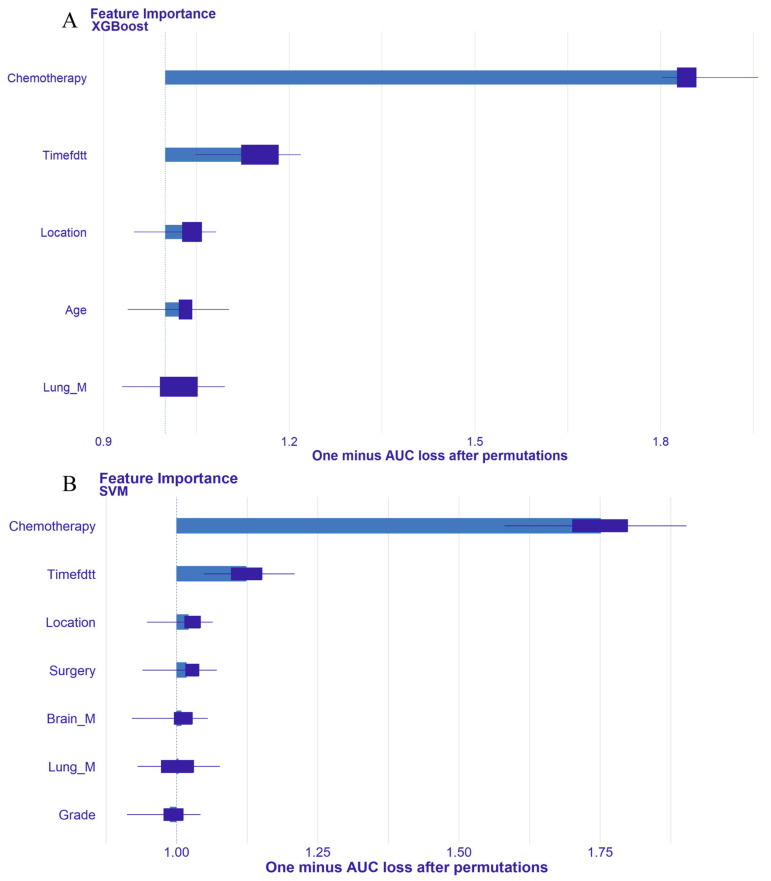
Feature importance ranking of XGBoost and SVM models. (**A**) Feature importance ranking of the optimal XGBoost model for predicting all-cause early mortality in esophageal cancer patients with liver metastasis. (**B**) Feature importance ranking of the optimal SVM model for predicting cancer-specific early mortality in esophageal cancer patients with liver metastasis.

**Figure 9:**
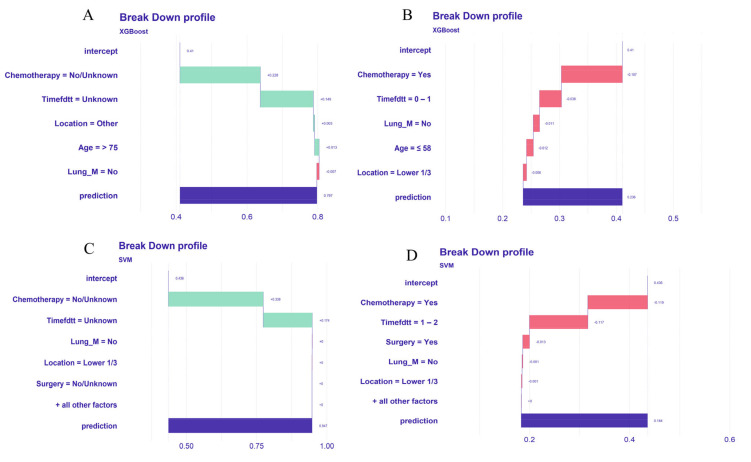
Interpretation of patient predictions in optimal models. (**A**) One patient with all-cause early death, (**B**) one patient with non-all-cause early death, (**C**) one patient with cancer-specific early death, (**D**) one patient with non-cancer-specific early death.

Using the DALEX package, we evaluated the importance ranking of variables in the optimal XGBoost and SVM models (Figure 8). The top three contributing variables were chemotherapy, time from diagnosis to treatment (in months), and primary tumor site. To enhance model interpretability, we further explored the impact of each variable on individual prediction results, visualizing which clinical features increased the prediction of early death and which decreased it. We selected four representative patients from the validation set to demonstrate individual early death prediction results (Figure 9). Red bars indicate a negative contribution to the prediction, while green bars indicate a positive contribution, displaying the predicted probability of early death. For example, Figure 9A shows a case of all-cause early death in an esophageal cancer liver metastasis patient (true positive). The XGBoost model’s baseline prediction value was 0.41. Factors such as not receiving chemotherapy (Chemotherapy = No/Unknown) (+ 0.228), unknown time from diagnosis to treatment (Timefdtt = Unknown) (+ 0.149), tumor location being other (+ 0.003), and age > 75 years (+ 0.013) positively influenced the prediction, while no lung metastasis (Lung_M = No) (−0.007) negatively influenced it. Considering other factors, the predicted probability of all-cause early death for this patient was 0.797.

## 4. Discussion

Esophageal cancer is the sixth leading cause of cancer-related deaths worldwide with a rising incidence and poor prognosis [27]. A major cause of mortality is distant metastasis, particularly to the liver. The long-term survival rate for metastatic esophageal cancer patients is very low with limited treatment options [28]. According to NCCN guidelines, these patients mainly receive palliative and supportive care [29]. Given these challenges, short-term survival prediction is essential for clinical decision making in cases of liver metastasis. However, few studies specifically address early mortality in this context.

This study explores the demographic and clinical characteristics of patients with liver metastasis from esophageal cancer, identifying risk factors for early death and predicting short-term survival for both all-cause and cancer-specific early death. We included patients with liver metastasis from esophageal cancer from the SEER database between 2010 and 2020 and found that 40% of metastatic EC patients experienced all-cause early death, indicating a need for heightened attention to this high rate of early mortality. Through feature selection, we identified five independent risk factors for all-cause early death in these patients: age, primary tumor location, chemotherapy, lung metastasis, and time from diagnosis to treatment. For cancer-specific early death, we identified seven independent risk factors: primary tumor location, tumor grade, surgical treatment, chemotherapy, brain metastasis, lung metastasis, and time from diagnosis to treatment. The primary tumor site, metastasis locations, and age have been widely demonstrated as prognostic factors associated with survival outcomes in metastatic patients. No significant association was found between marital status and cancer prognosis in this study, which may be attributed to differences in inclusion and exclusion criteria and sample sizes across studies [30].

Our study indicates that chemotherapy is an important protective factor against early death in esophageal cancer patients with liver metastasis. Currently, palliative chemotherapy and/or targeted therapy are considered standard treatments for this patient group, which supports the survival benefits observed in our analysis [31]. This finding is consistent with results from other related studies, which have similarly demonstrated the positive impact of chemotherapy on survival outcomes in metastatic cancer patients [32,33]. Additionally, surgery is necessary to improve short-term prognosis. Results indicate that patients with upper esophageal cancer have a higher likelihood of early death, potentially due to anatomical challenges, late-stage diagnosis, and squamous cell carcinoma histology [34,35]. Younger esophageal cancer patients have a higher risk of liver metastasis but a lower likelihood of early death compared to older patients. Age influences metastasis through various mechanisms, such as changes in the immune microenvironment, including inflammation and immunosenescence, which compromise extracellular matrix integrity [36,37]. This phenomenon aligns with other studies [38], indicating worse prognosis with increasing age. Lung and brain metastases are critical risk factors for early death with multiple metastases indicating advanced disease and poorer prognosis [39]. This underscores the aggressive nature of the disease and the impact of other metastatic sites on prognosis. Clinically, there is a need to prioritize the management and treatment of such aggressive metastatic diseases.

Using a large cohort and seven machine learning algorithms (LR, KNN, SVM, RF, XGBoost, LightGBM, and DT), we developed two accurate models to predict early death in liver metastasis from esophageal cancer patients. Compared to traditional logistic regression models, these sophisticated machine learning algorithms can utilize vast amounts of data for tasks such as risk stratification and survival estimation [40]. They offer flexibility, scalability, and superior model performance, as evidenced in multiple cancer studies [41]. Systematic evaluation identified XGBoost and SVM as the best models for predicting all-cause and cancer-specific early death, respectively, in liver metastasis from esophageal cancer patients.

Lastly, our study enhances model interpretability using the best-performing algorithms. Global interpretability was achieved by visualizing the importance ranking of variables within the optimal model, highlighting the key roles of chemotherapy, Timefdtt, and Lung_M in overall model prediction. For local interpretability, single-sample prediction decomposition was conducted to analyze individual prediction outcomes. This approach helps clinicians understand the impact of various variables on model predictions and provides rational explanations, aiding clinical decision making and targeted interventions [42]. For instance, in Figure 9A,C, the lack of chemotherapy significantly increases early death risk, while in Figure 9B,D, receiving chemotherapy significantly reduces this risk. Clinicians should prioritize providing chemotherapy to patients, barring clear contraindications. Moreover, the time from diagnosis to treatment is crucial; delays or unknown treatment times are associated with higher early death risks. Hence, clinical efforts should focus on minimizing this interval to reduce early mortality.

This study provides new insights into early death prediction for esophageal cancer patients with liver metastasis. Our models, particularly XGBoost and SVM, demonstrated strong predictive performance (AUC > 0.82), highlighting their potential for identifying high-risk patients in clinical settings, supporting close monitoring or early intervention. The predictive factors used in the models are easily understood and accessible in clinical practice, enhancing their practicality. Future research could consider incorporating additional factors closely related to early mortality, such as lifestyle factors, nutritional status, and detailed metastatic characteristics, to further improve model accuracy and expand clinical applicability. Integrating genetic and molecular markers may also help more accurately capture individual patient characteristics, optimizing prediction outcomes and supporting personalized treatment strategies.

This study has certain limitations. Firstly, as a retrospective analysis, it inherently carries potential biases. Secondly, we relied on data from the SEER database, which lacks detailed information on the number, volume, timing, and treatment of metastases. Additionally, this study lacks external validation data, and the sample size of patients who received surgical treatment is insufficient to draw definitive conclusions. Further research is needed to determine which patients might benefit from aggressive multimodal treatment.

## 5. Conclusions

This study identified risk factors associated with early mortality in patients with esophageal cancer liver metastasis (ECLM) and used seven machine learning algorithms to determine that XGBoost and SVM are the two optimal prediction models. The results showed that both models demonstrated high predictive performance in forecasting early mortality, providing valuable reference for clinical decision making and aiding in the optimization of individualized treatment for ECLM patients.

## Figures and Tables

**Table 1:** Clinical features and early death of patients with liver metastatic esophageal cancer.

Variables	Total ECLM (n = 1897)	No Early Death (n = 1138)	All-Cause to Early Death (n = 759)	Cancer-Specific Early Death (n = 717)
Age, n (%)				
≤58	526 (27.73)	353 (31.02)	173 (22.79)	162 (22.59)
59–75	1034 (54.51)	630 (55.36)	404 (53.23)	382 (53.28)
>75	337 (17.76)	155 (13.62)	182 (23.98)	173 (24.13)
Marital, n (%)				
Single	367 (19.35)	201 (17.66)	166 (21.87)	155 (21.62)
Married	1121 (59.09)	721 (63.36)	400 (52.7)	378 (52.72)
Wid/Div/Other	409 (21.56)	216 (18.98)	193 (25.43)	184 (25.66)
Race, n (%)				
White	1684 (88.77)	1017 (89.37)	667 (87.88)	633 (88.28)
Black	128 (6.75)	66 (5.8)	62 (8.17)	57 (7.95)
Other	85 (4.48)	55 (4.83)	30 (3.95)	27 (3.77)
Sex, n (%)				
Male	1627 (85.77)	971 (85.33)	656 (86.43)	624 (87.03)
Female	270 (14.23)	167 (14.67)	103 (13.57)	93 (12.97)
Location, n (%)				
Lower 1/3	1409 (74.28)	881 (77.42)	528 (69.57)	501 (69.87)
Middle 1/3	152 (8.01)	91 (8)	61 (8.04)	58 (8.09)
Upper 1/3	25 (1.32)	13 (1.14)	12 (1.58)	12 (1.67)
Other	311 (16.39)	153 (13.44)	158 (20.82)	146 (20.36)
Histologic, n (%)				
EAC	1467 (77.33)	930 (81.72)	537 (70.75)	506 (70.57)
ESCC	296 (15.6)	144 (12.65)	152 (20.03)	145 (20.22)
Other	134 (7.06)	64 (5.62)	70 (9.22)	66 (9.21)
Grade, n (%)				
G1/G2	1038 (54.72)	647 (56.85)	391 (51.52)	366 (51.05)
G3/G4	859 (45.28)	491 (43.15)	368 (48.48)	351 (48.95)
Stage_T, n (%)				
T1–T3	1363 (71.85)	827 (72.67)	536 (70.62)	506 (70.57)
T4	534 (28.15)	311 (27.33)	223 (29.38)	211 (29.43)
Stage_N, n (%)				
N0	502 (26.46)	274 (24.08)	228 (30.04)	221 (30.82)
N1–N3	1395 (73.54)	864 (75.92)	531 (69.96)	496 (69.18)
Surgery, n (%)				
No/Unknown	1863 (98.21)	1109 (97.45)	754 (99.34)	713 (99.44)
Yes	34 (1.79)	29 (2.55)	5 (0.66)	4 (0.56)
Radiotherapy, n (%)				
No/Unknown	1246 (65.68)	694 (60.98)	552 (72.73)	521 (72.66)
Yes	651 (34.32)	444 (39.02)	207 (27.27)	196 (27.34)
Chemotherapy, n (%)				
No/Unknown	613 (32.31)	120 (10.54)	493 (64.95)	466 (64.99)
Yes	1284 (67.69)	1018 (89.46)	266 (35.05)	251 (35.01)
Bone_M, n (%)				
No	1519 (80.07)	933 (81.99)	586 (77.21)	553 (77.13)
Yes	378 (19.93)	205 (18.01)	173 (22.79)	164 (22.87)
Brain_M, n (%)				
No	1821 (95.99)	1100 (96.66)	721 (94.99)	680 (94.84)
Yes	76 (4.01)	38 (3.34)	38 (5.01)	37 (5.16)
Timefdtt, n (%)				
0–1	735 (38.75)	514 (45.17)	221 (29.12)	208 (29.01)
1–2	522 (27.52)	406 (35.68)	116 (15.28)	108 (15.06)
>2	161 (8.49)	118 (10.37)	43 (5.67)	43 (6)
Unknown	479 (25.25)	100 (8.79)	379 (49.93)	358 (49.93)

Notes: 1. Wid/Div/Other in Marital is widowed, divorced, separated and unmarried or domestic partner; 2. other in Location is C15.9-Esophagus, NOS, C15.8-Overlapping lesion of esophagus, C15.1-Thoracic esophagus, C15.0-Cervical esophagus, C15.2-Abdominal esophagus; 3. Abbreviations: Timefdtt, time from diagnosis to treatment (month); EAC, esophageal adenocarcinoma; ESCC, esophageal squamous cell carcinoma. Bone_M, bone metastasis; Brain_M, brain metastasis; Lung_M, lung metastasis.

**Table 2:** Performance of the all-cause early death prediction model in patients with liver metastasis of esophageal cancer.

Data	Model	Accuracy	Sensitivity	Specificity	Precision	F1 Score	ROC_AUC	PR_AUC
Training set	Logistic	0.796	0.679	0.876	0.791	0.730	0.815	0.774
	DT	0.795	0.679	0.874	0.788	0.729	0.801	0.769
	KNN	0.783	0.693	0.844	0.754	0.722	0.816	0.787
	LightGBM	0.795	0.687	0.868	0.782	0.732	0.821	0.789
	RF	0.797	0.698	0.865	0.780	0.737	0.821	0.798
	XGBoost	0.796	0.660	0.890	0.804	0.725	0.811	0.777
	SVM	0.791	0.661	0.880	0.791	0.721	0.810	0.768
Validation set	Logistic	0.800	0.683	0.872	0.764	0.721	0.814	0.756
	DT	0.796	0.683	0.865	0.755	0.717	0.789	0.777
	KNN	0.769	0.678	0.824	0.701	0.689	0.771	0.718
	LightGBM	0.796	0.706	0.851	0.743	0.724	0.816	0.765
	RF	0.786	0.700	0.838	0.724	0.712	0.803	0.766
	XGBoost	0.809	0.667	0.895	0.795	0.725	0.822	0.772
	SVM	0.803	0.667	0.885	0.779	0.719	0.819	0.748

**Table 3:** Performance of the specific early death prediction model for patients with liver metastasis of esophageal cancer.

Data	Model	Accuracy	Sensitivity	Specificity	Precision	F1 Score	ROC_AUC	PR_AUC
Training set	Logistic	0.787	0.658	0.867	0.756	0.703	0.804	0.728
	DT	0.784	0.647	0.870	0.756	0.697	0.758	0.769
	KNN	0.782	0.663	0.856	0.742	0.700	0.804	0.736
	LightGBM	0.783	0.689	0.842	0.732	0.709	0.810	0.745
	RF	0.787	0.692	0.847	0.738	0.715	0.791	0.754
	XGBoost	0.780	0.663	0.853	0.737	0.698	0.795	0.734
	SVM	0.784	0.656	0.864	0.751	0.700	0.791	0.720
Validation set	Logistic	0.807	0.667	0.885	0.765	0.713	0.823	0.748
	DT	0.809	0.661	0.892	0.774	0.713	0.776	0.778
	KNN	0.788	0.661	0.859	0.724	0.691	0.776	0.701
	LightGBM	0.800	0.690	0.862	0.738	0.713	0.814	0.756
	RF	0.805	0.702	0.862	0.741	0.721	0.791	0.750
	XGBoost	0.800	0.684	0.866	0.741	0.711	0.834	0.767
	SVM	0.811	0.684	0.882	0.765	0.722	0.824	0.758

## Data Availability

These data can be found here: https://seer.Cancer.gov/ (accessed on 28 March 2024).

## Supplementary Materials

The following supporting information can be downloaded at https://www.mdpi.com/article/10.3390/life14111437/s1, Figure S1: Estimation of the appropriate cutoff value for age; Figure S2: Multicollinearity test; Figure S3: Heat map of variable correlation; Table S1: Parameters of the all-cause early death prediction model for patients with liver metastasis of esophageal cancer; Table S2: Parameters of the cancer-specific early death prediction model for patients with liver metastasis of esophageal cancer.
