# Supplementary material for: Prediction of Early Mortality in Esophageal Cancer Patients with Liver Metastasis Using Machine Learning Approaches
Source: Life (Basel). 2024 Nov 6;14(11):1437. doi: 10.3390/life14111437 (PMC11595315; doi:10.3390/life14111437)
Supplement: Supplementary file 1 [file life-14-01437-s001.zip › life-3268582-supplementary.pdf]

Supplementary Content

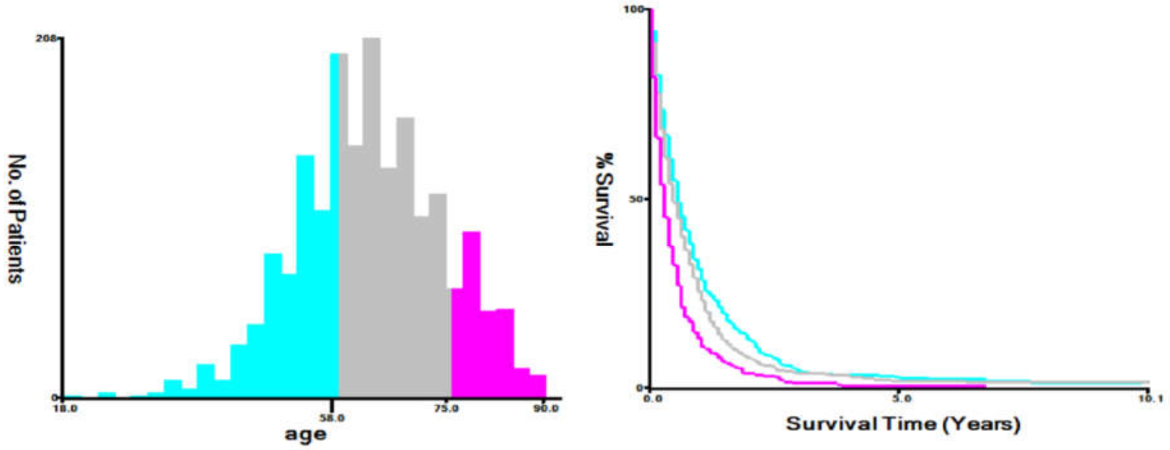

Figure S1. Estimation of the appropriate cutoff value for age.

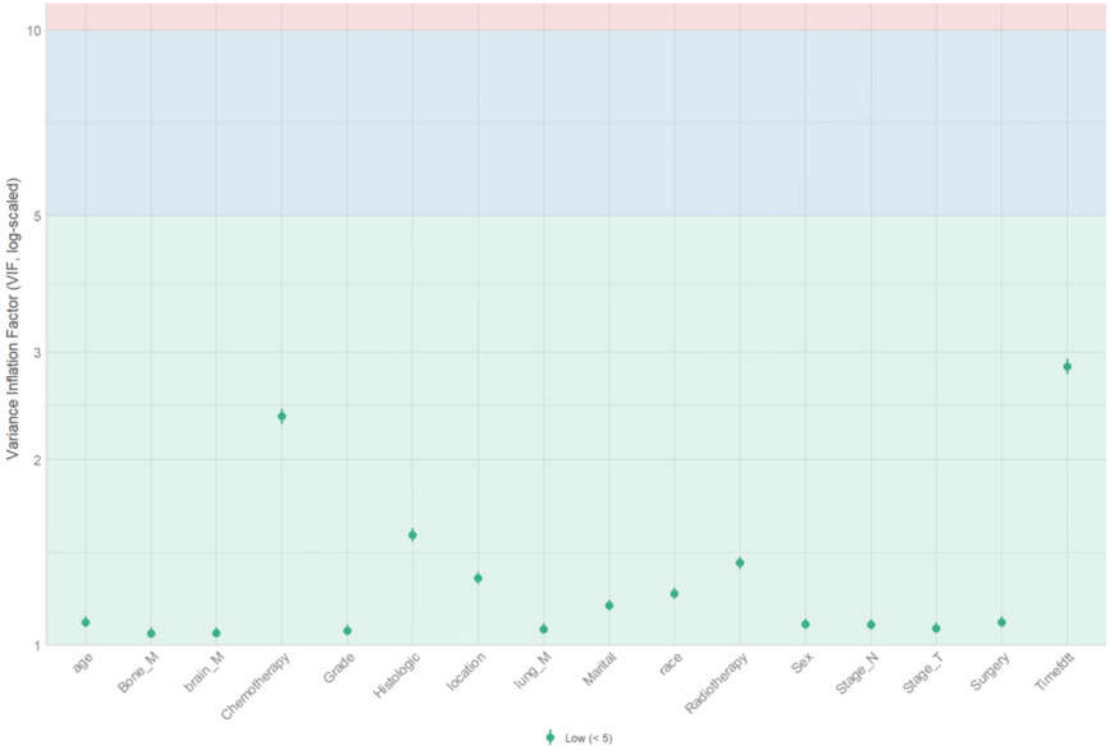

Figure S2. Multicollinearity test.

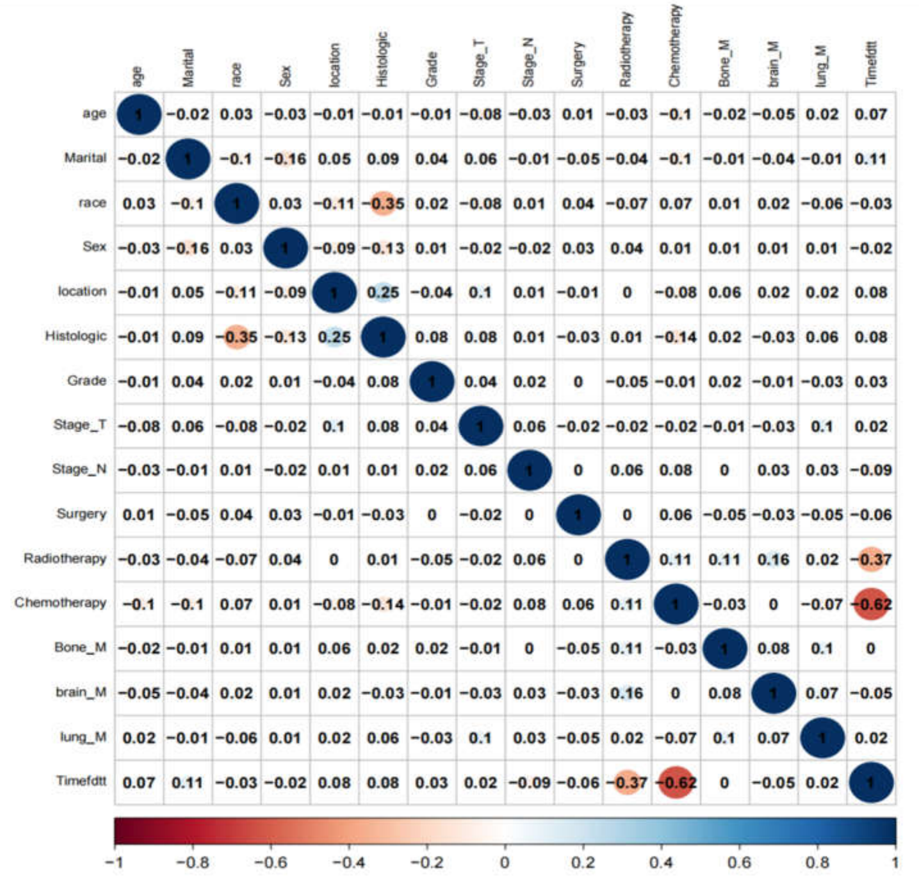

Figure S3. Heat map of variable correlation.

Generally, when the variance expansion factor (Variance Inflation Factor,VIF) is greater than 5 or 10, it indicates that the independent variable has a serious multicollinearity problem. The variable correlation heat map shows the Pearson correlation coefficient between variables, with color ranges from dark blue (indicating a high negative correlation, close to -1) to dark red (indicating a high positive correlation, close to 1). The middle is white (indicating no correlation, close to 0). The size of the circle also reflects the absolute value of the correlation coefficient, and the larger the circle, the stronger the correlation. All above prove that there is no serious multicollinearity problem in this study.(Figures S2, S3)

Table S1. Parameters of the All-cause Early Death Prediction Model for Patients With Liver Metastasis of Esophageal Cancer.

Approaches	Parameters
KNN	neighbors (k) = 11, weight_func = "rectangular", dist_power = 2.
RF	mtry = 49, n_estimators = 469, min_samples_leaf = 6.
DT	ccost_complexity = 0.00000833, tree_depth = 10, mtry = 40, min_n = 3.
LightGBM	mtry = 3, min_n = 10, trees = 362, tree_depth = 3, learn_rate = 0.00479, loss_reduction = 0.00994.
XGBoost	mtry = 2, min_n = 18, tree_depth = 2, learn_rate = 0.00342, loss_reduction = 0.00302, sample_size = 0.964, colsample_bytree = 0.8.
SVM	cost = 1, rbf_sigma = 0.0003.
Logistic	NA

Table S2. Parameters of the Cancer-specific Early Death Prediction Model for Patients With Liver Metastasis of Esophageal Cancer.

Approaches	Parameters
KNN	neighbors (k) = 6, weight_func = "rectangular", dist_power = 2.
RF	mtry = 7, n_estimators = 469, min_samples_leaf = 6.
DT	cost_complexity = 0.0223, tree_depth = 10, min_n = 3.
LightGBM	mtry = 6, min_n = 9, trees = 406, tree_depth = 5, learn_rate = 0.005, loss_reduction = 0.0963.
XGBoost	mtry = 2, min_n = 18, tree_depth = 2, learn_rate = 0.00342, loss_reduction = 0.00302, sample_size = 0.964.
SVM	cost = 0.12, rbf_sigma = 0.000153.
Logistic	NA
